# Optimization of dietary RNA interference delivery to western flower thrips *Frankliniella occidentalis* and onion thrips *Thrips tabaci*

**DOI:** 10.1002/arch.21645

**Published:** 2019-11-19

**Authors:** Awawing A. Andongma, Carolyn Greig, Paul J. Dyson, Natasha Flynn, Miranda M. A. Whitten

**Affiliations:** ^1^ Applied Molecular Microbiology Group, Swansea University School of Medicine Institute of Life Sciences Swansea UK; ^2^ Department of Physics, College of Science Swansea University Swansea UK; ^3^ School of Medicine, Faculty of Health Sciences University of Bristol Bristol UK

**Keywords:** diet modification, feeding dsRNA, onion thrips, western flower thrips, RNAi

## Abstract

In insect reverse genetics, dietary delivery of interfering RNAs is a practical approach in nonmodel species, such as thrips, whose small size, and feeding behavior restricts the use of other delivery methods. In a laboratory context, an unsuitable diet could confound the interpretation of an RNA interference (RNAi) phenotype, however well‐formulated artificial diets can minimize experimental variability, reduce the need for insect handling, and can further be used for roles, such as delivering double‐strand RNA (dsRNA)‐expressing recombinant bacteria. In this study, artificial diets for oral delivery of dsRNA were developed for two important pest thrips species, western flower thrips (*Frankliniella occidentalis*) and onion thrips (*Thrips tabaci*), with the goal of (a) stimulating feeding behavior, (b) supporting optimal growth rates of dsRNA‐expressing symbiotic bacteria, and (c) nutritionally supporting the thrips for sufficient periods to observe RNAi phenotypes. The efficacy of artificial diets for ingesting “naked” dsRNA or dsRNA‐expressing symbionts and dsRNA delivery via host plant uptake was evaluated. Compared with previously published diet formulations, new combinations based on tryptone, yeast, and soy were superior for enhancing feeding and longevity. However, simply adding “naked” dsRNA to an artificial diet was an unreliable form of RNAi delivery in our hands due to dsRNA degradation. Delivery via host plants was more successful, and the new diet formulation was suitable for symbiont‐mediated dsRNA delivery, which we believe is the most convenient approach for large‐scale knockdown experiments. This study, therefore, provides alternative methodologies for thrips rearing, dietary RNAi delivery, and insights into the challenges of performing dietary RNAi in nonmodel insects.

## Highlights

- Artificial diets developed to optimize feeding and survival in two thrips species are ideal for bacterial symbiont‐mediated dsRNA delivery.
- RNAi is also achieved via host plant dsRNA uptake, but thrips feeding activity can degrade naked synthetic dsRNA.

## Graphical abstract

A comparison of double‐strand RNA (dsRNA) oral delivery methods for two thrips species. We focus on diet formulations that enhance feeding activity and longevity and support bacterial symbiont‐mediated RNA interference, and compare with plant‐based dsRNA delivery and direct ingestion methods.

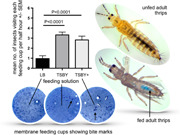

## 1 INTRODUCTION

Thrips (Thysanoptera: Thripidae) are major insect pests of ornamental crops and soft fruit and inflict mechanical damage arising from feeding and oviposition (Mouden, Sarmiento, Klinkhamer, & Leiss, 2017). Thrips also transmit devastating phytopathogens, such as the tomato spotted wilt virus (Goldbach & Peters, 1994). There are approximately six thousand thrips species, of which the western flower thrips (WFT) *Frankliniella occidentalis* (Pergande) and onion thrips *Thrips tabaci* (Lindeman), are particularly notorious due to their rapid spread across the globe (Kirk & Terry, 2003), multiple pesticide resistance and highly polyphagous behavior that makes them especially challenging to control (Mouden et al., 2017).

In the quest for effective management strategies for this pest, domestication of insects is imperative, and the diet of experimental insects is a key consideration. Indeed, the ability to domesticate insects for laboratory use has often hinged on artificial diets (Cohen, 2015), and this is especially true for nonmodel species. Thrips diet formulations have been the subject of several publications in the pregenomic era. In members of the family Thripidae, the options for an artificial diet formulation are limited by their sucking/piercing mouthpart anatomy, and feeding behaviors that rely mostly on plant sap (Hunter & Hsu, 1999; Hunter & Ullman, 1992). A pollen and honey diet for flower thrips was first developed (Murai & Ishii, 1982) as an alternative feeding and oviposition substrate, and modified over the years for various applications to meet the researcher's need. For example, Teulon and Penman (1991) changed the feeding/oviposition substrate to apple/peach juice or 10% sucrose solution and added mold inhibitors to support thrips’ viability for longer periods when oviposition was not a requirement. Similarly, a histidine–sucrose buffer solution was formulated by Hunter, Hsu, and Lawson (1995) to orally infect larval thrips with tospovirus without exposing them to toxins present in plants. Recently, a 3% sucrose solution has been used for the delivery of insecticide to the tobacco thrips (Huseth et al., 2016), dsRNA to onion thrips (Singh et al., 2019), and a 1.5% sucrose solution successfully delivered dsRNA to WFT (Jahani, Christiaens, & Smagghe, 2018).

The knockdown of essential genes through RNA interference (RNAi) offers an attractive control method for thrips and other insect pests, particularly as part of an integrated pest management approach, because it allows greater specificity than chemical insecticides (Whitten & Dyson, 2017; Whyard et al., 2015). One of the most efficient techniques used for the delivery of lethal double‐stranded RNA (dsRNA) is injection (Zotti & Smagghe, 2015). However, this is not a method of choice for thrips as high mortality rates have been reported after injection in WFTs (Badillo‐Vargas, Rotenberg, Schneweis, & Whitfield, 2015) and obviously unrealistic for large‐scale insect pest management programs. Ingestion of dsRNA can be costly, as continuous and repeated exposures are often required (Posiri, Ongvarrasopone, & Panyim, 2013; Whyard et al., 2015). Although the delivery of RNAi through dsRNA‐expressing transgenic crops has proven effective against certain pests, such as the western corn rootworm (Niu et al., 2017), this method is perceived as considerably more challenging against thrips species due to their polyphagous nature (Milne & Walter, 2000).

The practical choices for dietary RNAi delivery to thrips in a laboratory research setting are explored in this article, with an emphasis on optimizing phenotype assays and the screening of candidate genes. Several criteria must be considered and optimized when using an RNAi approach, such as the method of dsRNA synthesis (transcription efficiency, yield and quality, time, and overall cost), adequate nutritional support, the integrity of dsRNA before and after internalization by the insect (which is especially important for dietary delivery), and the relative ease with which phenotype assays can be set up and the insects manipulated. All these factors impact on the ultimate efficiency of knockdown and success of the RNAi strategy, and each may be more or less important depending on the intended application.

In recent years, the noninvasive symbiont‐mediated RNAi (SMR) strategy has been proven to be successful in thrips. SMR involves inoculating thrips with dsRNA‐expressing symbiotic bacteria (i.e., BFo2; “bacteria from *Frankliniella occidentalis* 2”) suspended in an artificial feeding solution (Whitten et al., 2016). The bacteria colonize the gut and continually synthesize dsRNA, which is a strategy that not only offers sustained RNAi from a single dose, but also potentially overcomes the problem of gut nuclease activity and could allow intraspecies horizontal and/or vertical transmission. The SMR technology also has potential to be employed especially in other nonmodel insects for the control of human disease vectors, agricultural pests and pathogens of beneficial insects.

In the case of symbiont‐delivered RNAi, artificial feeding solution is also the medium used to introduce the bacteria to the insect gut. A feeding solution based on diluted Luria broth (LB), sucrose, and NaCl was previously used to deliver dsRNA‐expressing symbiotic bacteria by re‐feeding to WFTs (Whitten et al., 2016). However, the usefulness of this formulation for nutritionally sustaining thrips over several days has not yet been tested since it was previously supplemented after 3 days with plant matter (runner beans). In our experience, it is preferable to maintain thrips on an artificial diet for the entire duration of an RNAi phenotype assay rather than feeding on plant material, as the latter is inherently more variable and it risks introducing foreign organisms such as mites or fungi coupled with the risk of losing experimental insects. Furthermore, for assessing phenotypes by RNAi, especially those which may take longer to manifest, a multi‐purpose diet is required that is both attractive and nutritionally supportive to thrips, and for SMR it must additionally support limited symbiont bacterial growth while preventing exponential growth that could result in septic shock of the host insect. It is also desirable to monitor feeding via the inclusion of a tracker dye that must be nontoxic both to the insect and its microbial flora.

In the current study, therefore, artificial diets were formulated as a key element in optimizing dietary delivery of RNAi in thrips *F. occidentalis* and *T. tabaci*. The relative merits of direct dsRNA feeding and vegetative delivery were also investigated and discussed with reference to the SMR technique already published by our lab (Whitten et al., 2016). The article also highlights possible challenges that could be faced using dietary RNAi delivery techniques.

## 2 MATERIALS AND METHODS

### 2.1 Insect rearing

The onion *Thrips tabaci* colony was established from adults collected from spring onions grown on a farm near Stratford‐on‐Avon, UK (grid reference: 52.158907, −1.917040) and subsequently reared on shop‐bought organic spring onions (*Allium fistulosum*) and leeks (*Allium ampeloprasum*), which were repotted for prolonged laboratory breeding. The insects were bred inside custom‐made caulked perspex cages or 4F series Bugdorms (MegaView Science Co., Ltd, Talchung, Taiwan). Lighting was supplied by equal numbers of red‐spectrum and blue‐spectrum 125 W compact fluorescent lamps (Greens Hydroponics, Bristol, UK).

The *Frankliniella occidentalis* (WFT) colony was established from adults and larvae collected from strawberry plants grown on a farm in Hereford, UK (grid reference: 51.952409,−2.652005). They were subsequently reared on supermarket‐purchased potted chrysanthemum plants and runner bean pods, neither of which was organic, in caulked perspex cages. Lighting was supplied using standard fluorescent tube ceiling lighting and/or biolamps (F.lli Della Marca s.r.l., Rome, Italy). Both insect species were reared at a temperature of 23°C ± 2°C, 60–70% relative humidity and a light:dark cycle of 14:10 hr.

### 2.2 Artificial diet feeding set‐up

A simple membrane‐feeding apparatus was used to feed thrips with artificial diet solutions. Feeding cups were made from the lids of sterile 1.5 ml microfuge tubes, filled with approx 300 µl feeding solution and covered with stretched alcohol‐sterilized Parafilm M (Bemis Co., Inc., Neenah) with which the solution was in full contact. Feeding cups and experimental insects were placed in 50 × 24 mm flat‐bottom glass tubes (Regina Industries Ltd, Newcastle, UK) with plastic stoppers into which a filter pipette tip was inserted for ventilation. Unless otherwise stated, 20 insects (10 larvae, 10 adults) were used for each experiment with at least three biological replicates.

### 2.3 Development of insect artificial diets

Different feeding solutions were formulated to compare (a) the survival and growth of symbiotic BFo2 bacteria (ultimately intended to express dsRNA *in insecta*), (b) thrips feeding preferences, (c) thrips survival, and (d) suitability for longer‐term phenotype assays. The main reagents used in these experiments were sourced as follows: Yeast extract: Melford Laboratories Ltd, Ipswich, UK; LB and sucrose: Sigma‐Aldrich Company Ltd, Gillingham, UK; tryptone soy broth: Formedium^TM^, Hunstanton, UK. The assessed diets were as follows:

(1) Apple juice and honey diets: Sterile solutions of 10% (wt/vol) pressed apple juice (Copella Fruit Juices Ltd, Boxford, UK) and 10% (wt/vol) honey were individually assessed in preliminary experiments but abandoned due to rapid bacterial and fungal colonization that affected insect survival within 24 hr (data not shown).
(2) LB diet: A LB‐based feeding solution consisting of 20% (vol/vol) LB, 2.4% (wt/vol) sucrose, and 0.32% (wt/vol) NaCl (Whitten et al., 2016).
(3) Tryptone soy broth and yeast extract (“TSBY”) diet: LB was replaced by 0.2X tryptone soy broth with 1 g/L yeast extract to provide a greater range of peptones and B vitamins.
(4) TSBY with supplements (“TSBY+”) diet: A more complex TSBY‐based diet intended mainly for onion thrips, with 0.01% (wt/vol) wheatgerm oil. Unlike WFTs that feed mainly on the flower (Atakan, Coll, & Rosen, 1996; Gill, Garg, Gill, Gillett‐Kaufman, & Nault, 2015), onion thrips are attracted to young plant shoots that contain high ascorbic acid concentrations (Dias, 2019). Therefore TSBY+ diet also included 0.1% (wt/vol) ascorbic acid, and when fed to onion thrips (but not WFTs), this feeding solution also included 1% (wt/vol) crushed organic leek juice (*Allium ampeloprasum*) as an additional feeding stimulant.

All the above dietary ingredients were filter sterilized, and when required, 0.03% (wt/vol) methylene blue tracker dye (Sigma‐Aldrich Co., Ltd) was also incorporated that was visible through the living cuticle, to easily identify fed insects. This dye had already been confirmed as nontoxic both to the insects and the symbiotic bacterium BFo2 (data not shown; see Whitten et al., 2016 and below). A wide range of candidate tracker dyes were tested in preliminary experiments (data not shown) including food dyes and neutral red (based on its effectiveness in aphids; Bilgi, Fosu‐Nyarko, & Jones, 2017). However, the viability of the symbiont was not sufficiently maintained in any of the alternative dyes tested.

### 2.4 BFo2 symbiotic bacteria growth in different feeding solutions

We previously demonstrated that BFo2, a bacterial symbiont of WFT (De Vries, Van der Wurff, Jacobs, & Breeuwer, 2008; Facey et al., 2015), can be used to successfully deliver RNAi to these insects via an apramycin‐resistant dsRNA‐expressing BFo2 clone (Whitten et al., 2016). In the current study, preliminary experiments demonstrated that a GFP‐expressing clone of BFo2 is able to colonize onion thrips guts for at least 3 days (data not shown). BFo2 was therefore the bacterium of choice to deliver dsRNA to both onion and WFTs. To assess BFo2 growth in each feeding solution, the LB, TSBY, and TSBY+ (with leek) diets were inoculated with a final concentration of 1–2 × 10^7^/ml BFo2 (expressing dsRNA; Whitten et al., 2016) and incubated for 72 hrs in feeding cups. Colony forming units (CFUs) of BFo2 were determined at time zero and after 72 hrs by inoculating onto LB‐sucrose agar (with apramycin antibiotic selection) and incubating at 30°C for 2 days.

### 2.5 Thrips feeding solution preferences

A Petri dish containing onion thrips and three feeding cups (one for each of the three diet solutions) was set up to determine feeding preferences. The feeding activities of onion thrips were monitored by time‐lapse photomicroscopy using a DinoLite AM2111 USB microscope (AnMo Electronics Corporation, Taipei, Taiwan). Seventeen hours of footage were recorded over four observation periods, during the three‐day experiment. The feeding cup membranes were also examined for puncture marks indicating the number of feeding events.

### 2.6 Thrips survival with different feeding solutions

Insect survival rates were estimated by counting the number of live thrips after defined time periods with experimental feeding solutions as the only nutritional source. An acceptable survival rate is considered essential for RNAi assays to ensure any phenotypes observed are due to gene knockdown and not poor diet. For the WFTs, 21 glass vials were set up per diet, each containing 20 adults and larvae. Three glass vials were opened per diet treatment per day for 7 days, and the total numbers of live and dead insects were counted and then killed. The survival of WFTs fed on water, TSBY and TSBY+ were compared with LB diet. For the onion thrips, 15–30 adults and larvae in each of three vials were assessed per feeding solution at 2–3 days and 6–7 days. In this case, LB diet was compared with TSBY+ inoculated with 1.25 × 10^7^/ml GFP‐expressing BFo2 to determine whether the reintroduction of such concentrations of symbiotic bacteria would have an impact host insect survival.

### 2.7 dsRNA synthesis

The template for dsRNA synthesis was obtained by two different methods. A 500 base pair fragment of the *F. occidentalis vATPase‐B* sequence (previously reported by (Badillo‐Vargas et al., 2015) was commercially synthesized and cloned into the mammalian gene expression vector VB181204‐1167mqb (map https://en.vectorbuilder.com/vector/VB181209‐1063eux.html) flanked by restriction sites and 5′ T7 promoters. This target is an essential gene whose knockdown in WFT is lethal. The linearized plasmid was then used as a template for transcription reactions, with the sense and antisense reactions transcribed in separate tubes then annealed in equimolar concentrations. The plasmid method was complemented by a polymerase chain reaction (PCR)‐based approach in which *F. occidentalis* total RNA was extracted using TRIzol reagent (Thermo Fisher Scientific, Waltham, MA) and then reverse‐transcribed to complementary DNA (cDNA) using an ultraScript 2.0 cDNA synthesis kit (PCR Biosystems Ltd, London, UK). The cDNA template for dsRNA synthesis was amplified by end‐point PCR with vATPase‐B specific primers Forward: 5′‐GGACCTTTGGTGATTTTGGA‐3′ and Reverse: 5′‐AACACAGATTTGCCTGGGAC‐3′. Control dsRNA was a *Streptomyces coelicolor agarase dagA* fragment (primers in Whitten et al., 2016). Each primer included a T7 RNA polymerase promoter sequence at the 5′ end: GAATTAATACGACTCACTATAGGGAGA.

Purified amplicon (~1–2 µg) was then used to synthesize dsRNAs using the Megascript RNAi synthesis Kit (Thermo Fisher Scientific), with the forward and reverse reactions in the same tube. It is noteworthy that we found it important to avoid the use of mercaptoethanol in the clean‐up of dsRNA because even residual amounts appeared to be toxic to the insects.

### 2.8 Comparing different oral dsRNA delivery methods in WFTs

We compared the relative efficacy of target gene knockdown in WFTs using different oral RNAi delivery methods: Ingestion of lab‐synthesized “naked” dsRNA via membrane‐feeding, and ingestion of lab‐synthesized dsRNA via host plant feeding (vegetative delivery) to compare with the proven SMR method (Whitten et al., 2016).

Synthetic dsRNA was added to TSBY feeding solution (the optimal for WFTs; see Results) at a final concentration of 0.5 µg/µl and fed to adult and larval thrips using the feeding cup system described above. Surprisingly, these preliminary experiments in fact revealed no significant phenotypes (data not shown). To determine whether this was due to dsRNA degradation and establish the possible cause, four feeding cups were incubated as follows: dsRNA diluted in TSBY exposed to thrips feeding, dsRNA in TSBY without insect feeding, dsRNA only without TSBY, and TSBY only to serve as the negative control. The cups were kept in an incubator under the rearing conditions described above. After 2, 3, 4, and 6 days, the dsRNA in the cup contents was visualized on a 1% agarose gel.

### 2.9 Synthetic dsRNA delivery via vegetable delivery

The petiole of a fresh runner bean plant leaf was immersed in 0.3 µg/µl of dsRNA and 1% tracking dye solution (supermarket‐purchased E133 brilliant blue food coloring) for 2 hours at room temperature, during which uptake of dsRNA by transpiration could be visualized as the food dye colored the leaves. About 1000 WFTs (larva and adults) were added into each perspex cage containing the leaves. After 18 hr under normal insectary conditions, insects (10 larvae, 10 adults) that had taken up dsRNA (confirmed by the presence of blue dye in their gut) were counted into a glass tube and subsequently fed on the TSBY diet. Thrips mortality was estimated by counting the insects after 2, 3, 4, and 6 days (and killed after counting). Each dsRNA treatment had three biological replicates and each biological replicate (different leaves) had three technical replicates (*n *= 720 insects per target gene).

## 3 RESULTS

### 3.1 BFo2 symbiotic bacterial growth in different feeding solutions

**Figure 1:**
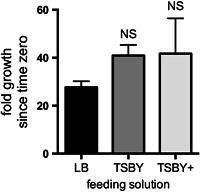
Growth rates of dsRNA‐expressing BFo2 symbiotic bacteria are not significantly (NS) different in the three feeding solution formulations, as measured by fold increase in CFUs over 72 hr. Bar = *SEM*. (Note: the TSBY+ solution used in this experiment contained leek extract). CFU, colony forming unit; dsRNA, double‐strand RNA; *SEM*, standard error of mean; TSBY, tryptone soy broth and yeast extract

All the feeding solutions supported the survival of dsRNA‐expressing BFo2 bacteria. The mean fold increase in BFo2 CFUs over 72 hrs was 27.7× when grown in the LB diet. In comparison, TSBY and TSBY+ (and leek extract) supported slightly higher growth (40.9× and 41.7× respectively), however these changes were not statistically significant (one‐way analysis of variance [ANOVA] with Dunn's multiple comparisons test; Figure 1). These growth rates were therefore considered acceptable and unlikely to result in septic shock after ingestion.

### 3.2 Onion thrips feeding solution preferences

**Figure 2:**
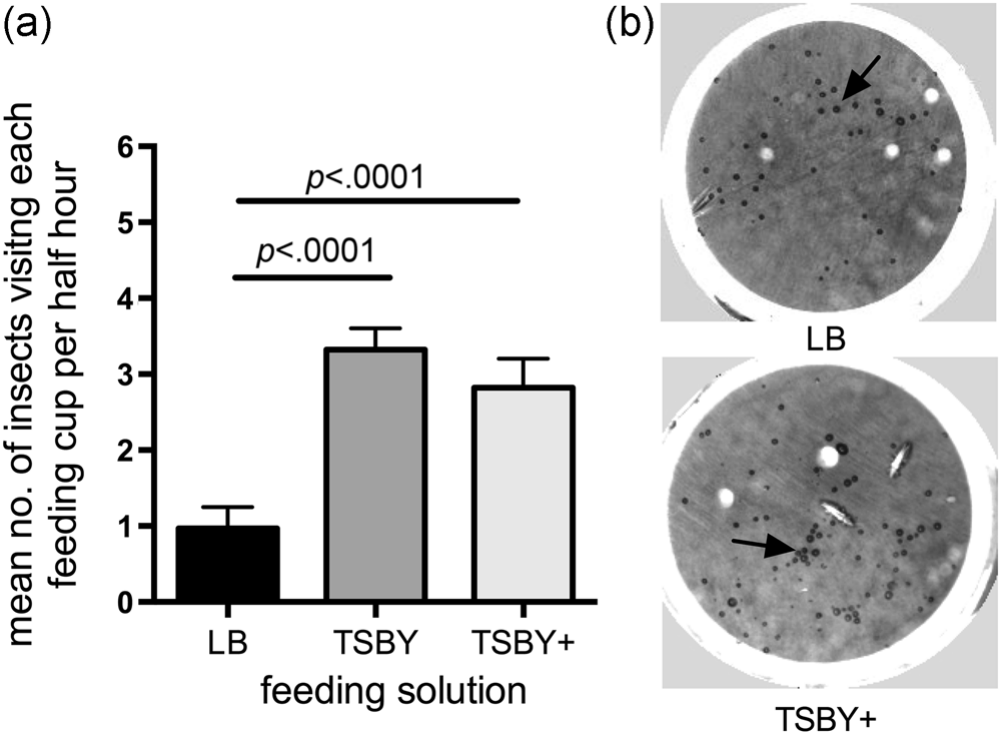
(a) Onion thrips show a distinct preference for TSBY and TSBY+ feeding solutions compared with the LB‐based diet, as demonstrated by the average number of insects visiting feeding cups. Data gathered by time‐lapse video microscopy at intervals over three days. Bar = *SEM*. (b) Examples of puncture marks on the feeding cup membranes (arrows). LB, Luria broth; *SEM*, standard error of mean; TSBY, tryptone soy broth and yeast extract

Compared with the original feeding LB Solution, TSBY and TSBY+ were both markedly more attractive to onion thrips (Figure 2). The TSBY feeding cup was visited 3.7 times more frequently and the TSBY+ was visited four times more frequently than LB (both *p* < .001, Friedman's test with Dunn's multiple comparisons test). Approximately twice as many puncture holes were made in the TSBY and TSBY+ cup membranes. Interestingly, there was no significant difference in onion thrips’ preference between the TSBY and TSBY+ diets. However, since TSBY+ successfully supported the growth of BFo2 (Figure 1), we favored this feeding formulation for onion thrips in subsequent experiments as we expected the leek extract to assist in suppressing the growth of contaminant microbes introduced by the thrips feeding apparatus. This is because *Allium* extracts are known to have antimicrobial properties (Kyung, 2012).

### 3.3 WFTs and onion thrips survival with different feeding solutions

**Figure 3:**
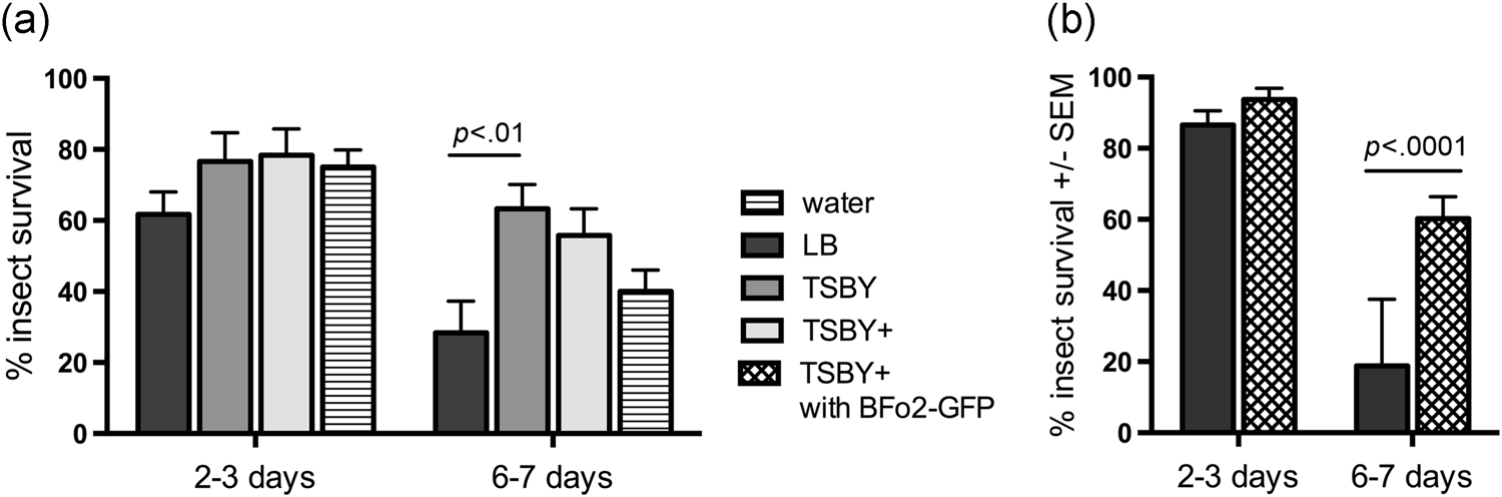
Thrips survival on various experimental feeding solutions measured over periods of 2–3 days and 6–7 days. Combined data for equal numbers of larvae and adults. (a) Western flower thrips (3 biological replicates, 60 insects per treatment per timepoint); (b) onion thrips (2–3 biological replicates, 44–87 insects per treatment per timepoint). Bar = *SEM. SEM*, standard error of mean

For the WFTs, none of the diets significantly altered insect survival by the 2–3 day time period. However, by 6–7 days, the TSBY diet supported the continued survival of significantly more insects compared with LB (Figure 3a; 63.3% survival compared with 28.3% respectively; *p* < .01; one‐way ANOVA with Sidak's multiple comparisons test). It should be noted that the 6–7 day period is sufficient time in which to observe lethal RNAi phenotypes (Whitten et al., 2016). The other diets (water and TSBY+) yielded intermediate results that were not significantly different from the LB diet (Figure 3a). Surprisingly, insects fed with water lived at least as long as those on the LB diet. Similarly, after 2–3 days, the onion thrips that had fed on TSBY+ inoculated with GFP‐expressing BFo2 bacteria evidenced a 93.7% survival rate compared with 86.5% survival among LB‐fed onion thrips and 88.9% of TBSY+ fed insects (Figure 3b; no significant difference). By days 6–7, only 18.8% of the LB‐fed onion thrips remained alive, compared with 60.2% of the TSBY+ plus GFP‐BFo2+ leek extract (Figure 3b).

### 3.4 Feeding lab‐synthesized dsRNA to the WFTs is ineffective

**Figure 4:**
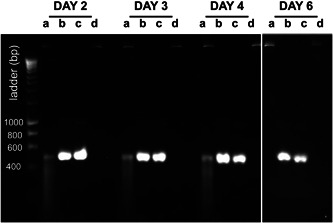
dsRNA in feeding cups is rapidly degraded by western flower thrips feeding activity. a = TSBY with dsRNA and with thrips feeding; b = TSBY with dsRNA without thrips; c = dsRNA (positive control) without thrips; and d = TSBY with thrips feeding (negative control). dsRNA, double‐strand RNA; TSBY, tryptone soy broth and yeast extract

Feeding “naked” lab‐synthesized dsRNA to the WFTs proved to be unsuccessful inasmuch as the expected mortality phenotype was not evidenced (data not shown). We therefore investigated whether the integrity of the dsRNA had been compromised, and found that significant degradation occurred within 48 hr and by day six all of the dsRNA had been completely degraded (Figure 4). In contrast, there was no dsRNA degradation in the feeding cups containing TSBY plus dsRNA and dsRNA only, i.e., without insect feeding activity (Figure 4) demonstrating that the sterile TSBY diet had no effect on dsRNA integrity. This strongly indicates that thrips feeding activity in some way was the cause of the dsRNA degradation.

### 3.5 dsRNA delivery to the WFTs by leaf feeding

**Figure 5:**
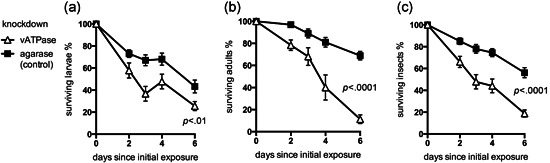
The effectiveness of dsRNA knockdown by vegetative delivery of lab‐synthesized dsRNA. Survival of (a) larval and (b) adult western flower thrips after ingesting plant material that had previously taken up dsv*ATPase‐B* or control ds*agarase*; (c) combined data for adults and larvae. Bar =* SEM*. dsRNA, double‐strand RNA; *SEM*, standard error of mean

There was a significant decrease in the survival of WFT larvae that fed on bean leaf containing lab‐synthesized ds*vATPase‐B*, compared with the control ds*agarase* (Figure 5). By Day 6, 68.9% of control adults were still alive compared with just 11.3% of the vATPase‐B knockdowns (*p* < .0001, unpaired *t* test), and 25.6% of *vATPase‐B* knockdown larvae compared with 43.3% of the control larvae (*p* < .01, unpaired *t* test; Figure 5). This suggests lethal dsRNA delivery by the vegetative method is effective against WFTs.

## 4 DISCUSSION

The purpose of this article is to update the state‐of‐the‐art on dsRNA oral delivery to thrips and to enable the reader to make an informed choice of the most efficacious methods for their intended RNAi application, especially for nonmodel insects. The diet is extremely important in maintaining insect health and should ideally not be a source of variability when conducting a phenotype assay. Options include feeding lab‐synthesized dsRNA on its own, or via a food plant, and artificial diets can eliminate the risks and variability associated with plant diets, such as unexpected outbreaks of predators or fungal growth. When dsRNA is delivered by recombinant symbiotic bacteria, such as BFo2 (Whitten et al., 2016), it is also important that the diet supports the desired growth characteristics of the symbionts.

For both onion thrips and WFTs, we found that tryptone soy broth based enriched diets (TSBY) offered significant improvements in insect feeding rates and survival over the LB‐based diet, and this was particularly true for experiments lasting longer than 3 days. It is, however, interesting that even water can support the survival of insects for a few days, and in fact water and sucrose has been exploited in dsRNA delivery to thrips (Jahani et al., 2018; Singh et al., 2019). The notion that simple diets can also support thrips development is corroborated by Jahani et al. (2018), in which a 1.5% sucrose diet allowed the full post‐embryonic development of WFTs. However, nutrient‐limited diets should be used with caution and could be a reason for the incidences of cannibalism seen in Stage 2 WFTs larvae (Jahani et al., 2018).

In the current study, the final optimized onion thrips feeding solution used for SMR phenotype assays was thus TSBY+, which is based on diluted tryptone soy broth, yeast extract, wheatgerm oil, ascorbic acid, and (for onion thrips) crushed leek juice. This diet significantly enhanced the overall percentage of thrips that fed during a 3‐day BFo2 “oral inoculation window” (approximately four times more than the original LB‐based formulation). Although we included leek extract as an antibacterial (that nevertheless does not affect the viability of the symbiont BFo2; Figure 1), the extract does little to enhance feeding behavior and is not strictly necessary. The TSBY+ diet also better supported the thrips’ nutritional requirements but without significantly altering the growth dynamics of the bacterial inoculum, and it is in turn also possible that the BFo2 bacteria in the diet help support thrips survival. It is very important, however, to avoid excessive bacterial overgrowth to prevent mortality associated with septic shock that could confound analysis of the RNAi phenotype. The growth rate of the BFo2 bacteria was not significantly different in TSBY+ relative to the LB‐based diet.

Plant‐mediated delivery of lab synthesized dsRNA (via transpiration into the leaf and subsequent feeding) was relatively successful on a laboratory scale, but the expense of synthesizing dsRNA for field applications is likely to be prohibitive. Transgenic dsRNA‐expressing crops would most likely be effective with the thrips species in the current study, but with the caveat that as polyphages, thrips could still thrive on other plants in the local vicinity. Similarly, feeding dsRNA directly via the artificial feeding solution is usually not scalable, and in our hands degradation of ds*vATPase‐B* was surprisingly rapid under such conditions. However, it should be noted that good dsRNA stability with an almost identical ds*vATPase‐B* fragment has been achieved in a simpler artificial feeding solution consisting of 1.5% sucrose with food coloring (Jahani et al., 2018). Nevertheless our data indicate that thrips feeding activity can in some way cause dsRNA degradation rather than the feeding solution per se. The mechanism of dsRNA degradation in our experiments is unknown but we hypothesize the presence of RNases in *F. occidentalis* fluids, which are known to contribute to the variation of RNAi efficacy in different insects species (Singh, Singh, Mogilicherla, Shukla, & Palli, 2017). In the Lepidoptera for example, nonspecific nucleases in the gut lumen are responsible for dsRNA breakdown (Jing & Han, 2014; Liu, Smagghe, & Swevers, 2013; Liu, Swevers, Iatrou, Huvenne, & Smagghe, 2012) and in the hemipterans, salivary secretions, and body fluids are responsible for dsRNA degradation (Allen & Walker III, 2012; Christiaens, Swevers, & Smagghe, 2014; Singh et al., 2017). An alternative source of RNase could be microbes on the insect mouthparts, introduced to the dsRNA solution via mechanical inoculation. Experiments to understand these mechanisms in thrips will be an area for further research.

The efficiency of dsRNA synthesis and the ease of delivery to nonmodel insects, in particular, have a significant impact on the final choice of technique to be exploited for research and/or as part of a pest management strategy. With respect to dsRNA delivery methods, the current study suggests that vegetative delivery is more efficient than directly feeding naked dsRNA. Vegetative delivery has also been shown to be effective in Hemiptera (Ghosh, Hunter, Park, & Gundersen‐Rindal, 2017), and is an acceptable alternative to bacteria‐mediated delivery. Symbiont‐mediated delivery (Whitten et al., 2016), however is an extremely convenient approach especially when combined with the TSBY modified diet that avoids the inherent risks and extra insect handling associated with using plant material and offers the additional potential advantages of long‐term knockdown and transmission between insects.

## CONFLICT OF INTERESTS

The authors declare that there are no conflict of interests.
